# Maternal nutrient restriction in late pregnancy programs postnatal metabolism and pituitary development in beef heifers

**DOI:** 10.1371/journal.pone.0249924

**Published:** 2021-04-08

**Authors:** John M. Long, Levi A. Trubenbach, Kenneth C. Hobbs, Andrew E. Poletti, Chelsie B. Steinhauser, Jane H. Pryor, Charles R. Long, Tryon A. Wickersham, Jason E. Sawyer, Rhonda K. Miller, Rodolfo C. Cardoso, Michael Carey Satterfield

**Affiliations:** 1 Department of Animal Science, Texas A&M University, College Station, Texas, United States of America; 2 Department of Veterinary Physiology and Pharmacology, Texas A&M University, College Station, Texas, United States of America; 3 King Ranch^®^ Institute for Ranch Management, Texas A&M University – Kingsville, Kingsville, Texas, United States of America; University of Illinois, UNITED STATES

## Abstract

Maternal undernutrition during pregnancy followed by *ad libitum* access to nutrients during postnatal life induces postnatal metabolic disruptions in multiple species. Therefore, an experiment was conducted to evaluate postnatal growth, metabolism, and development of beef heifers exposed to late gestation maternal nutrient restriction. Pregnancies were generated via transfer of *in vitro* embryos produced using X-bearing sperm from a single Angus sire. Pregnant dams were randomly assigned to receive either 100% (control; n = 9) or 70% (restricted; n = 9) of their total energy requirements from gestational day 158 to parturition. From post-natal day (PND) 301 until slaughter (PND485), heifers were individually fed *ad libitum* in a Calan gate facility. Calves from restricted dams were lighter than controls at birth (*P*<0.05) through PND70 (*P*<0.05) with no difference in body weight from PND105 through PND485 (*P*>0.10). To assess pancreatic function, glucose tolerance tests were performed on PND315 and PND482 and a diet effect was seen with glucose area under the curve being greater (*P*<0.05) in calves born to restricted dams compared to controls. At slaughter, total internal fat was greater (*P*<0.05) in heifers born to restricted dams, while whole pituitary weight was lighter (*P*<0.05). Heifers from restricted dams had fewer growth hormone-positive cells (somatotrophs) compared to controls (*P*<0.05). Results demonstrate an impaired ability to clear peripheral glucose in heifers born to restricted dams leading to increased deposition of internal fat. A reduction in the number of somatotrophs may contribute to the adipogenic phenotype of heifers born to restricted dams due to growth hormone’s known anabolic roles in growth, lipolysis, and pancreatic islet function.

## Introduction

Worldwide demand for protein continues to grow as land and other resources become increasingly scarce. These accelerating trends have turned producers’ attention to implementing confinement, or semi-confinement feeding in an attempt to increase production per unit area of land, and improve animal nutrient utilization by reducing feed intake [1, 2]. Semi-confinement feeding allows for a high degree of control over nutritional management and affords producers the potential opportunity to significantly improve the efficiency of nutrient utilization in beef cows. Previous work has found that when high quality feedstuffs are limit-fed, cow nutrient requirements are lower and diet digestion is greater than what is predicted by the National Research Council (NRC) [1, 3]. These findings support the concept of increased efficiency of nutrient utilization and represent a beneficial mechanism that could be of potential use to economically improve beef cow management. However, if this reduction in dietary intake does not truly meet the combined maintenance requirements of maternal tissue and pregnancy, the developing fetus may be subject to inappropriate metabolic programming relative to its extra-uterine environment. If this were to occur, any economic gains made during management of pregnant females would subsequently be lost due to poor growth and performance of their offspring.

Offspring phenotype is the product of inherited genes and the environment in which those genes are developed. Fetal programming is the concept that expression or function of inherited genes is altered by external stimulus during pre- and perinatal development [4]. This phenomenon has been observed in livestock species in response to maternal nutrient restriction during pregnancy with effects on fetal growth as well as skeletal muscle and pancreas function [5–8]. Additionally, maternal nutrient restriction during gestation has been shown to decrease postnatal growth rates in sheep progeny [9–11], pig progeny [12], and cattle progeny [13, 14]. Furthermore, a number of cattle studies have reported reductions in economically important carcass traits such as hot carcass weight (HCW), yield grade, quality grade, and meat tenderness in response to maternal undernutrition [14–16]. Many of these studies have observed impaired steer progeny performance in a feedlot setting [14, 17], and inefficient heifer progeny reproductive performance [18, 19].

The anterior pituitary gland produces hormones that regulate growth (growth hormone, GH), metabolic function (thyroid-stimulating hormone, TSH), stress response (adrenocorticotropic hormone, ACTH) and reproductive function (luteinizing hormone, LH; follicle-stimulating hormone, FSH; prolactin, PRL). Previous studies in sheep have shown that maternal nutrient restriction during the first half of gestation alters the proportion of somatotropes within the fetal anterior pituitary [20] and, in post-natal life, enhances the ACTH/cortisol response during stress as well as decreases plasma concentrations of progesterone in females [21]. The impact of maternal nutrient restriction on the anterior pituitary in cattle has not been studied, but does have the potential to help explain alterations in growth, metabolism, and reproductive performance that have been previously described in nutrient-restricted offspring. Therefore, we hypothesized that a moderate maternal nutrient restriction of total energy during the second half of pregnancy would decrease birthweight and growth efficiency of offspring through impaired metabolic and pituitary function.

The objective of this study was to investigate the effect of maternal nutrient restriction on postnatal growth efficiency by monitoring body weight (BW), ribeye area (REA), last rib backfat (LRBF), and glucose tolerance as well as feed intake and efficiency through the weaning, growing, and finishing stages of production. Upon slaughter, the effects of maternal nutrient restriction during pregnancy were assessed on carcass traits and organ development.

## Materials and methods

All experimental procedures were approved by the Institutional Animal Care and Use Committee of Texas A&M University (AUP#2014–0005; AUP#2014–0236; AUP#2017–0253).

### Embryo production and transfer

Embryos were produced *in vitro* (IVP) as previously described [5, 22]. Briefly, oocytes were collected from Angus-based abbatoir-derived ovaries (DeSoto Bioscience, Seymour, TN) and were fertilized with semen, sexed for X-bearing sperm, from a single Angus sire after 22–24 h of maturation. Matured oocytes and sperm were co-incubated in BO-IVF (IVF Bioscience, Cornwall, UK) for 16–18 h at 38.5°C, in a 5% CO_2_ in air humidified incubator (IVF = Day 0). Presumptive zygotes were cleaned of cumulus cells and placed in BO-IVC (IVF Bioscience) medium covered in oil (Irvine Scientific, Santa Ana, CA) at 38.5°C, in a 5% CO_2_/ 5% O_2_/ 90% N_2_ humidified incubator for six days.

On day 7 post-IVF, only grade I or II blastocysts or compacted morulae were manually split in 75 μl splitting medium (Vetoquinol, Pullman, WA) under microscopic conditions using a micro-blade (Shearer Precision Products, Pullman, WA, USA) to generate monozygotic twins. Embryo halves (demi-embryos) were washed, loaded in Vigro^™^ Holding Plus medium (Vetoquinol) in ¼ ml straws, covered with a metal-tipped sheath and chemise (PETS, Canton, TX, USA) and singularly transferred non-surgically using a Cassou gun to synchronized recipient virgin dams of Angus-based composite breed-type uniform in age, body condition and frame score (n = 72). Pregnancy was confirmed by ultrasound on gestational day (GD) 60. Due to having only a few instances where both embryo halves established a pregnancy, calves used in this study are all half-siblings, but not monozygotic twins. All pregnancies with monozygotic twins were used in a different study [5], therefore, 21 pregnant recipients were enrolled in this study.

### Experimental design

Pregnant recipients were assigned to receive either 100% (control; n = 9) or 70% (restricted; n = 9) of their total energy requirements according to standard beef cattle nutritional models [3] from GD 158 through parturition (Table 1). The restriction to 70% of NRC predicted requirements was chosen based on observations that restriction to 80% during mid to late gestation had minimal effect on maternal energy retention due to apparent adaptation in maintenance demand [1, 2]. Requirements were estimated per individual based on body weight, and each individual was provided a prescribed daily ration to achieve treatment levels of energy intake (1.54 Mcal/kg NEm = 100%). The diet was formulated such that individuals fed 70% of the diet still met or exceeded protein requirements and the diet was fortified with a mineral/vitamin package to meet or exceed requirements for animals in both treatment groups. Recipients were housed (3 or 4 per pen) in concrete surfaced pens with two thirds of the pen covered by a roof and equipped with automatic watering points. Each pen was equipped with multiple Calan gate feeders (American Calan, Northwood, NH), which allowed for cows to be housed in small groups, but fed individually. Maternal BW was assessed every 14 days, while rib eye area (REA) and last rib back fat (LRBF) were recorded every 28 days by ultrasonography.

**Table 1:** Formulated ingredient and nutrient composition of diet fed to dams from gestational day 158 to 265.

*Ingredient*	*% As fed*
Wheat straw	34.52
Cracked corn	29.46
Dried distiller’s grain	27.46
Urea	1.10
Molasses	5.00
Mineral	2.46
*Diet components* ^a^	*DM basis* ^b^
CP, %	16.30
ME, Mcal/kg	2.45
NE_m_, Mcal/kg	1.54
*Chemical Composition*	*DM basis* ^b^
CP, %	12.7
OM, %	92.7
ADF, %	26.6

^a^ According to National Research Council (NRC) model estimates

^b^ Dry matter (DM) contents = 83.42%

Of the 21 recipients enrolled in the study, two gave birth to bull calves and one heifer calf died at two days old, therefore, those recipients were excluded from the study analysis. After parturition, dams and their heifer calves were maintained on pasture that met 100% of their nutrient requirements for the duration of lactation. Heifer weights were collected at birth, then at 35-day intervals until slaughter at postnatal day (PND) 485. Two weeks after weaning (PND 210), heifers were placed in a grower facility and fed a high percentage roughage grower ration. While in the grower facility heifers were trained to use the Calan gate feeders and from PND 305 to 315 heifers were transitioned on to the finishing ration that would be provided for the duration of the feed trial.

### Postnatal feed trial

Beginning at PND 315, feed was provided *ad libitum* and daily intake was monitored to determine average daily gain and gain to feed ratio. Body weights were recorded weekly throughout the trial. Heifer REA and LRBF were determined via ultrasound at weaning (PND 210), initiation of the feed trial (PND 315), and prior to slaughter (PND 420). Two heifers were humanely euthanized prior to end of study (PND 462 and 469) due to injuries incurred during study, one heifer from the control group and one heifer from the restricted group.

### Glucose tolerance test

At PND 301 and 482, heifers were subjected to an intravenous glucose tolerance test (IVGTT) performed as previously reported [23]. Heifers were fitted with an indwelling jugular cannula and blood samples were collected prior to dextrose infusion to provide baseline glucose values. Dextrose was infused (50% wt/vol) at 0.5 mL/kg BW (0.25 g/kg BW) immediately following baseline sample. After completion of dextrose infusion, blood samples were collected at 2.5, 5, 7.5, 10, 15, 30, 60, 90, and 120 min, in 10-mL tubes containing sodium-heparin (Monoject, Sherwood Medical, St. Louis, MO), placed on ice for 30 min, and centrifuged at room temperature for 7.5 min at 2500 × *g*. Plasma was aliquoted and stored at -20°C. Plasma glucose concentrations from all GTT time points were determined using a colorimetric glucose assay kit (catalog no. STA-680; Cell Biolabs, Inc., San Diego, CA). The intra- and inter-assay variations were 2.6% and 10.1%, respectively. The area under the curve (AUC) was calculated using the trapezoidal summation method and results are presented in (μg/ml)*min.

### Tissue collection and handling following necropsy

Prior to slaughter on PND 485, heifer BW was recorded. Heifers were then intravenously administered phenytoin/pentobarbital at 0.20 mg/kg BW (Beuthanasia-D, Merck Animal Health, Madison, NJ) to effect. Immediately following euthanasia administration, organs (brain, whole pituitary, heart, lungs, liver, pancreas, spleen, kidneys, adrenals, ovaries, uterus, small intestine, rumen, internal fat, gastrocnemius muscle) were removed and weights were recorded. Samples from each organ (samples were collected from the interior of each organ) were preserved in either 4% paraformaldehyde (2 cubes of approximately 1 cm^3^ each) for 24 hours before being stored in 70% ethanol for histological analyses or snap frozen in liquid nitrogen (3 tubes of 1 g per tube) and stored at -80°C for future molecular biology analyses. At the time of slaughter, rib eye area (REA), 12th rib fat thickness, kidney/pelvic/heart fat (KPH), and marbling score were determined. A 2.54 cm slice of longissimus dorsi muscle was removed from the 13^th^ rib, immediately refrigerated, vacuum-packaged, and stored at 2°C for 10 days prior to evaluation for Cook Yield and Warner-Bratzler shear force as previously described [24]. Briefly, muscle samples were cooked to an internal temperature of 70°C, then allowed to cool to room temperature before six cores samples from each animal were subjected to shearing. Cook yield (%) was determined by dividing the cooked sample weight by the raw sample weight.

### Immunohistochemistry

A series of immunohistochemistry assays were performed to characterize the number and distribution of the different cell types in the anterior pituitary. The peptides growth hormone (GH), adrenocorticotropic hormone (ACTH), prolactin (PRL), thyroid stimulating hormone beta subunit (TSHβ), and luteinizing hormone beta subunit (LHβ) were immunolocalized in bovine pituitary glands as previously described [25]. Briefly, paraffin-embedded pituitary glands were sectioned at 5 μm thick, placed on slides, deparaffinized, and run through series of ethanol washes. Antigen retrieval was performed using protease for ACTH, PRL, TSHβ, and LHβ, while no antigen retrieval was necessary for GH. The primary antibodies, rabbit anti-sheep GH antiserum (1:1000), rabbit anti-human ACTH antiserum (1:1000), rabbit anti-sheep PRL antiserum (1:1500), rabbit anti-sheep TSHβ antiserum (1:100), and rabbit anti-sheep LHβ antiserum (1:1500), were obtained from Dr. A. F. Parlow at the National Hormone and Peptide Program, National Institute of Diabetes and Kidney Disease and were incubated with sections overnight at 4°C. The negative control was 2% normal rabbit serum (31883; Invitrogen, Carlsbad, CA) diluted to the same concentration as the corresponding antiserum. Immunoreactive peptides were visualized using the Vectastain Elite ABC Kit (PK-6101; Vector Laboratories, Inc., Burlingame, CA) according to the manufacturer’s instructions with 3,3′-diaminobenzidine tetrahydrochloride (DAB; D5637; Sigma-Aldrich) as the color (brown) substrate. Sections were counterstained with Harris-modified hematoxylin (SH30-500D; Fisher Scientific, Waltham, MA), and coverslips were affixed using Permount mounting medium (SP15-100; Fisher Scientific).

Cell counts were performed using bright-field illumination with a 100X objective under oil (Nikon Eclipse 80i; Nikon Corp.; Tokyo, Japan). One hundred nuclei were counted in each of four representative sections for a total of 400 cells per animal. A single observer performed all counts with any cell containing brown DAB stain considered positive for the peptide of interest. Cell counts of positive cells were analyzed and reported as a ratio of positive cells to the total number of cells counted.

Color images were captured using bright-field illumination (Nikon Eclipse Ni-E; Nikon Corp) with a 40X objective. All images were captured at the same threshold settings and size, then saved as .tif images for analysis of DAB intensity as previously described [26]. Briefly, images were subjected to an analysis of the color histogram from a background section of the image to determine similar RGB values for all images (ImageJ 1.43j, National Institutes of Health, Bethesda, MD) The “color deconvolution” plug-in was applied using the HDAB setting to separate the hematoxylin and DAB into different panels. From the DAB image, the mean intensity was calculated to yield a final DAB intensity where a higher value equals darker staining. Four images of only the anterior pituitary were captured per animal (total area of 2.15 mm^2^) and the mean intensity was averaged.

### Statistical analysis

Maternal BW is reported as the weight of the maternal tissue without the fetus and associated tissues/fluids and was calculated as:

ActualMaternalBW−GravidUterus=NetMaternalBW

The gravid uterus weight was calculated using the following formula [27]:

$$GravidUterus(kg)=[(BirthWeight(kg)x19.32)(e^{\left(0.02xGD)-(0.0000143{xGD}^{2}\right)})]/1000$$

Data were analyzed using MIXED procedures in SAS 9.3 (SAS Inst. Inc., Cary, NC). Maternal BW, LRBF, and REA were analyzed using repeated measures as were offspring BW, LRBF, and REA. Main effects (diet, day) and the interaction (diet by day) were included in the model. The subject was specified as animal within treatment. Offspring carcass characteristics and organ weights were subjected to a Students’ t-test. Glucose parameters (AUC, Cmax, Tmax) were subjected to MIXED procedures in SAS with the main effects (diet, day), the interaction (diet by day), and the subject (animal within treatment) included in the model. Cell counts were analyzed using GLIMMIX procedures in SAS with the main effect of treatment and the subject as animal within treatment. Means are reported as LSMeans ± SEM with *P* ≤ 0.05 considered significant.

## Results

**Fig 1:**
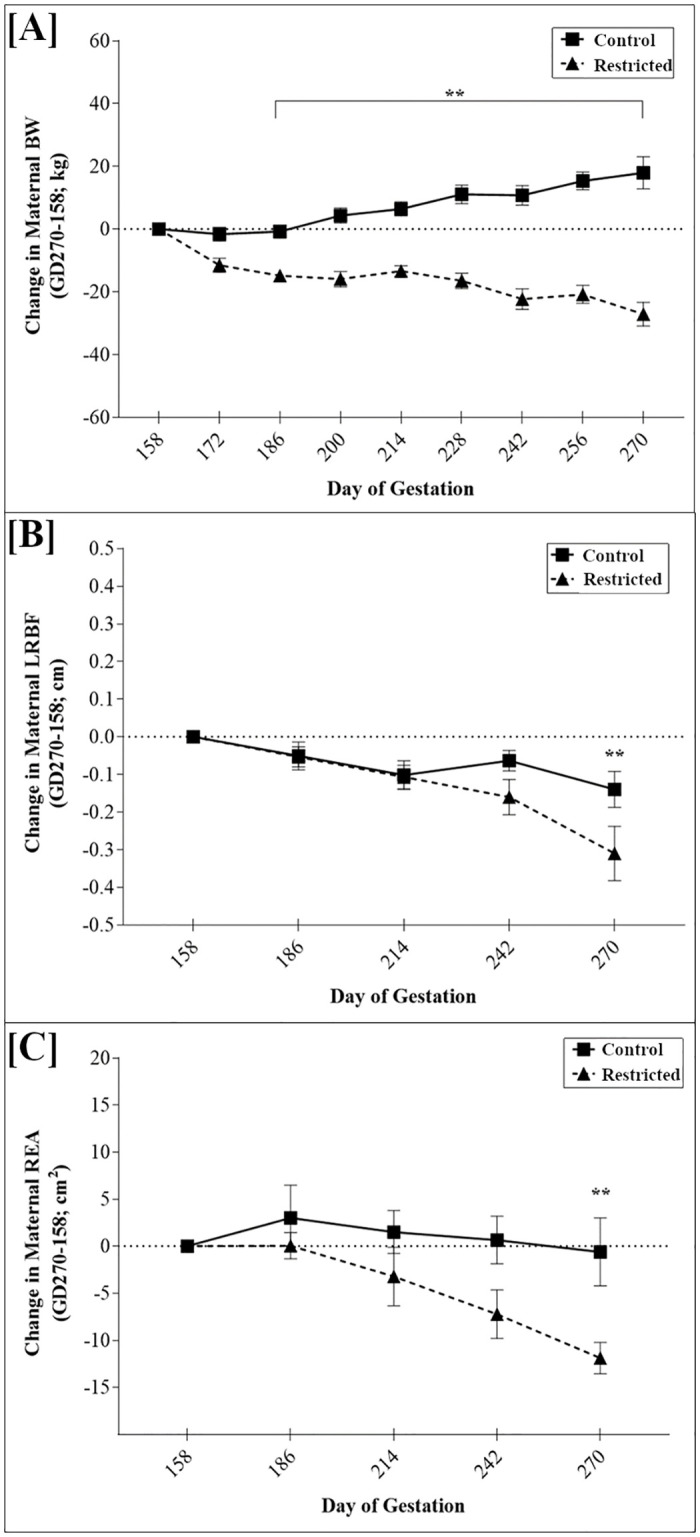
Effects of feeding 100% NRC (control; n = 9) or 70% NRC (restricted; n = 9) energy requirements to pregnant beef heifers from gestational day 158 to parturition on maternal [A] net body weight (BW;**P<0.01), [B] last rib back fat (LRBF; **P<0. 01), and [C] ribeye area (REA; **P<0. 01).

A reduction in net maternal BW was observed (*P*<0.01) by GD 186 in nutrient-restricted dams and continued to GD 270, while control dams increased in BW (Fig 1A). Maternal LRBF decreased in both groups, but the change was greater in the restricted dams at GD 270 (Fig 1B; *P*<0.01). Maternal REA was reduced in both groups, but only at GD 270 was REA significantly decreased in restricted dams (*P*<0.01) compared to control dams (Fig 1C).

Calves born to control dams had heavier birthweights than calves born to nutrient-restricted dams (*P* = 0.04; Table 2). Body weight continued to be greater in calves from control-fed dams compared to restricted dams through PND 70 (*P*<0.05; Table 2). There was no difference in heifer BW between groups from PND 105 through PND 485 (*P*>0.10; Table 2).

**Table 2:** Effects of maternal nutritional energy restriction (100% vs 70%) from GD 158 to parturition on post-natal offspring body weight (kg) in Angus cross heifers (n = 9 per treatment).

Day	Treatment		SE	*P*-value
	*Control*	*Restricted*		
PND 0	39.1	32.4	2.1	**0.04**
PND 35 ± 3	64.9	53.2	2.5	**<0.01**
PND 70 ± 3	105.9	90.6	5.2	**0.05**
PND 105 ± 3	151.7	136.8	6.4	0.12
PND 140 ± 3	195.8	184.2	7.7	0.30
PND 175 ± 3	239.5	222.4	8.4	0.17
PND 210 ± 3	269.4	252.8	10.0	0.26
PND 245 ± 3	283.3	268.4	11.2	0.36
PND 280 ± 3	324.9	311.4	11.8	0.43
PND 315 ± 3	347.4	337.7	13.0	0.61
PND 350 ± 3	403.9	389.0	16.3	0.53
PND 385 ± 3	454.0	431.0	17.5	0.37
PND 420 ± 3	475.6	477.3	15.4	0.94
PND 455 ± 3	522.0	504.6	17.0	0.48

GD, gestational day; PND, post-natal day

There was no effect of maternal diet on calf REA at PND 210, 315 or 420 (*P*>0.10; Table 3). Similarly, there was no effect of maternal diet on calf LRBF at PND 210, 315, or 420 (*P*>0.10; Table 3). There was no effect of maternal diet (*P*>0.10) on feed intake, total BW gain, or gain to feed ratio during *ad libitum* feeding from PND 315 to PND 485 (Table 4).

**Table 3:** Effects of maternal nutritional energy restriction (100% vs 70%) from GD 158 to parturition on REA and LRBF of Angus cross offspring fed *ad libitum* from PND 315 to PND 485 (n = 9 per treatment).

	Treatment		SE	*P*-value^1^
	*Control*	*Restricted*		
REA (cm^2^)				
PND 210	52.6	49.9	2.6	0.45
PND 315	65.6	63.9	3.2	0.70
PND 420	88.9	79.9	3.5	0.08
LRBF (cm)				
PND 210	2.89	2.77	0.10	0.39
PND 315	2.90	2.90	0.06	0.96
PND 420	3.30	3.36	0.06	0.48

GD, gestational day; PND, post-natal day; REA, ribeye area; LRBF, last rib back fat

**Table 4:** Effects of maternal nutritional energy restriction (100% vs 70%) from GD 158 to parturition on feed intake and feed efficiency of Angus cross offspring fed *ad libitum* from PND 315 to PND 485 (n = 8 per treatment).

	Treatment		SE	*P*-value
	*Control*	*Restricted*		
Initial BW (kg)	342.9	328.4	8.6	0.33
Final BW (kg)	532.4	516.0	12.6	0.45
Total BW gain (kg)	189.5	187.6	9.4	0.94
Overall ADG (kg/d)	1.11	1.10	0.05	0.94
Total Intake (kg)	1591.4	1482.8	57.4	0.32
Intake (kg/d)	9.47	8.82	0.34	0.33
Intake (% mean BW)	2.15%	2.07%	0.04	0.39
Gain: Feed, total	0.119	0.129	0.007	0.39

GD, gestational day; PND, post-natal day; BW, body weight; ADG, average daily gain

Intravenous glucose tolerance tests (IVGTT) conducted at PND 301 and 482 demonstrated that calves born to restricted dams had greater area under the curve (AUC; *P*<0.05) compared to calves born to control fed dams (Table 5). Maximum concentration of plasma glucose (Cmax; μg/ml) in response to infusion was not different between treatments, nor was time to maximum concentration of plasma glucose (Tmax; min) in response to infusion (Table 5).

**Table 5:** Effects of maternal nutritional energy restriction (100% vs 70%) from GD158 to parturition on glucose tolerance of Angus cross offspring at initiation (PND 301; n = 9 per treatment) and end (PND 482; n = 8 per treatment) of *ad libitum* feed trial.

	Age	Treatment		SE	*P*-value		
		*Control*	*Restricted*		*Trt*	*Age*	Trt*Age
Glucose AUC (μg/ml)*min	301	1,606,906	1,620,842	44,391	**<0.05**	0.06	0.14
	482	1,458,555	1,602,036				
Cmax (μg/ml)	301	22,844	19,899	2,219	0.68	0.18	0.33
	482	17,686	18,996				
Tmax (min)	301	3.25	4.06	0.46	0.28	0.42	0.42
	482	3.25	3.35				

GD, gestational day; PND, post-natal day; AUC, area under the curve; Cmax, maximum plasma concentration of glucose; Tmax, time to maximum plasma concentration of glucose

Organ weights collected from heifer calves on PND 485 are presented in Table 6. The pituitary gland was heavier in calves born to control-fed dams compared to those from restricted dams (*P*<0.05). Further, heifer calves born to control-fed dams possessed less internal fat (*P*<0.05) when compared to their restricted counterparts. There was no difference (*P*>0.10) in weights of any other organs assessed at slaughter between maternal dietary treatments.

**Table 6:** Effects of maternal nutritional energy restriction (100% vs 70%) from GD 158 to parturition on organ weights of Angus cross offspring at PND 485 (n = 16).

	Treatment		SE	*P*-value
	*Control*	*Restricted*		
Brain (g)	343.92	357.17	11.62	0.43
Whole Pituitary (g)	2.45	2.09	0.12	**0.04**
Heart (kg)	1.98	1.85	0.08	0.25
Lungs (kg)	2.82	2.66	0.14	0.45
Liver (kg)	7.65	8.00	0.42	0.56
Pancreas (g)	261.02	281.68	23.95	0.55
Spleen (kg)	1.73	1.77	0.14	0.84
Kidneys (kg)	1.25	1.20	0.09	0.67
Adrenals (g)	24.74	23.31	1.88	0.59
Ovaries (g)	15.79	19.19	2.06	0.26
Uterus (g)	138.86	143.96	13.04	0.79
Small Intestine (kg)	8.02	8.49	0.56	0.56
Rumen (kg)	20.36	19.33	0.89	0.41
Total Internal Fat (kg)	19.02	23.22	1.28	**0.04**
Gastrocnemius Muscle (kg)	1.64	1.54	0.07	0.29

GD, gestational day; PND, post-natal day

Parameters indicative of carcass quality, including quality grade, REA, and 12^th^ rib fat thickness did not differ in response to maternal diet during gestation (*P*>0.10; Table 7). Additionally, parameters of cutability, including Warner-Bratzler shear force of the longissimus dorsi muscle did not differ between treatment groups (*P*>0.10; Table 8).

**Table 7:** Effects of maternal nutritional energy restriction (100% vs 70%) from GD 158 to parturition on carcass quality of Angus cross offspring fed *ad libitum* from PND 315 to PND 485 (n = 16).

	Treatment		SE	*P*-value
	*Control*	*Restricted*		
Fat Thickness (cm)	0.75	0.71	0.08	0.75
REA (cm^2^)	89.0	81.7	5.0	0.32
Numerical Marbling^1^	448	435	30	0.77
Quality Grade^2^	408	400	14	0.68

GD, gestational day; PND, post-natal day; REA, ribeye area

^1^ 400 = small^00^; 500 = modest^00^

^2^ 400 = choice; 500 = prime

**Table 8:** Effects of maternal nutritional energy restriction (100% vs 70%) from GD158 to parturition on carcass shear and cook yield of Angus cross offspring fed *ad libitum* from PND 315 to PND 485 (n = 16).

	Treatment		SE	*P*-value
	*Control*	*Restricted*		
Cook Time (min)	16	14	1.2	0.43
Cook Yield (%)	82.5	82.9	1.6	0.85
Warner-Bratzler shear force (kg)	3.38	2.78	0.34	0.23

*GD, gestational day; PND, post-natal day

**Fig 2:**
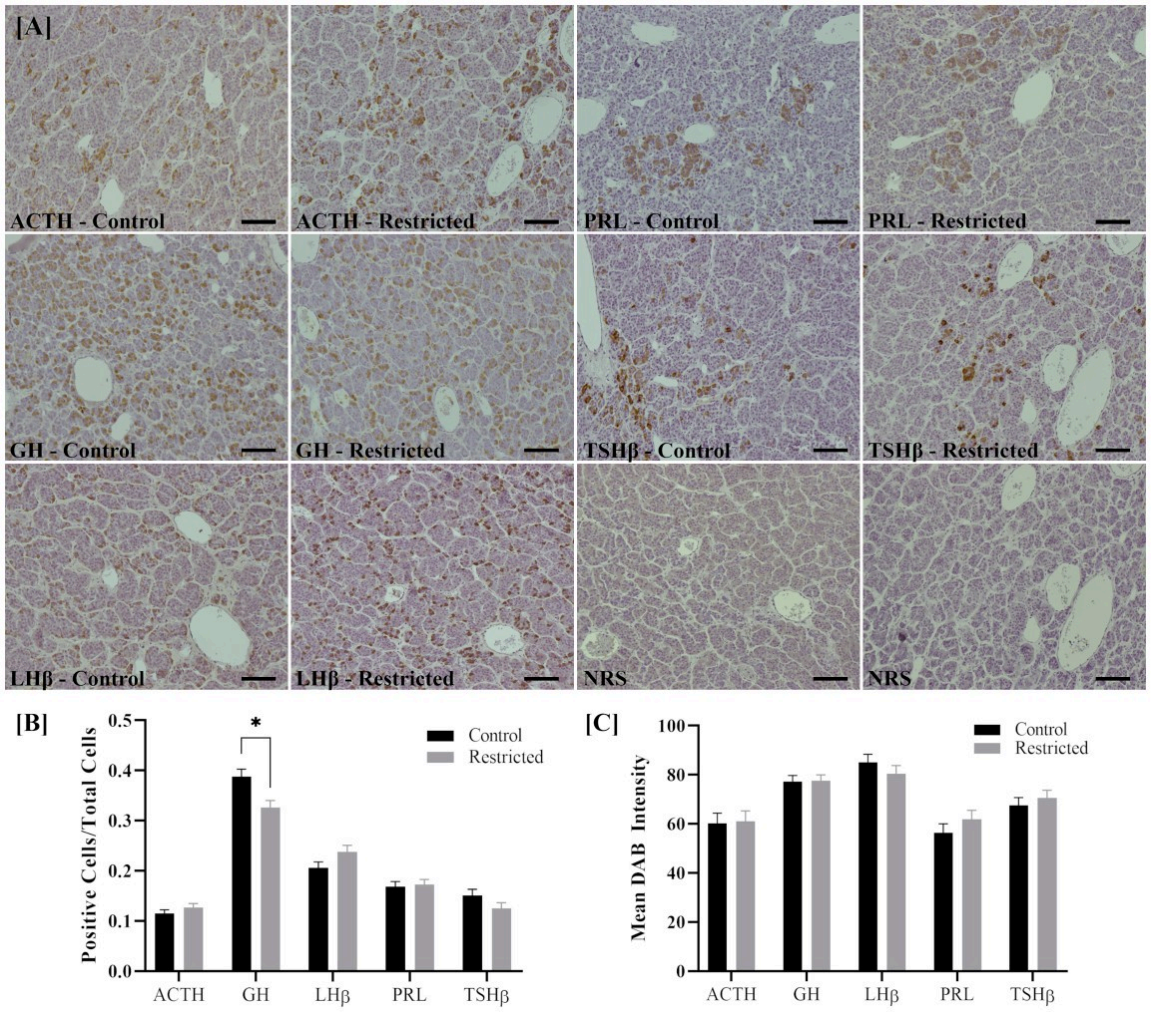
Immunohistological localization **[A]** of ACTH, GH, LHβ, PRL and TSHβ in the pituitary of offspring from mothers fed 100% NRC (control; n = 8) or 70% NRC (restricted; n = 8) energy requirements from gestational day 158 to parturition. The percentage of cells immuno-stained for GH in the pituitary was greater (*P<0.01) in heifers from control-fed dams as compared to restricted [**B**], with all other hormones showing no difference. There were no differences in staining intensity between control and restricted animals for any of the hormones [**C**]. The NRS panel represents the negative control. Scale bar is 100 μm. Values are presented as means ± SEM with *P*<0.05 considered significant. Legend: ACTH, adrenocorticotropic hormone; GH, growth hormone; LHβ, beta unit of luteinizing hormone; PRL, prolactin; TSHβ, beta unit of thyroid-stimulating hormone; NRS, normal rabbit serum.

Immunohistochemical localization of ACTH, GH, LHβ, PRL, and TSHβ peptides within the pituitary are depicted in Fig 2A. The percentage of GH-positive cells, somatotrophs, out of 400 cells counted per animal was greater in pituitaries of calves born to control dams compared to restricted dams (*P*<0.01; Fig 2B). There was no difference in intensity of staining for any of the peptides between treatment groups (*P*>0.10; Fig 2C).

## Discussion

A growing body of evidence highlights the link between maternal nutritional and environmental exposures during pregnancy and associated alterations in the postnatal phenotypes of the offspring. More specifically, it has been suggested in mammalian species that in response to maternal malnutrition the fetus will alter its metabolism to preferentially store consumed calories as fat for future utilization during periods of hardship rather than for the deposition of lean tissue mass [28–30]. Despite these observations, limited data exists in the bovine species regarding the effects of a moderate total energy nutritional restriction during late gestation on postnatal growth and performance. Results of the present study highlight a reduction in birth and postnatal BW to PND 105, after which weights did not differ. There were no observed differences in REA, LRBF or feed efficiency. Consistent with observations from sheep [10], we observed an increase in abdominal fat at PND 485 in calves born to restricted dams. We also observed a decrease in the weight of the whole pituitary gland and number of somatotrophs in heifers born to restricted dams.

Phenotype of the offspring in response to an environmental insult, such as maternal nutritional restriction, is dependent upon a variety of factors including timing of the insult, duration of the insult, severity or dosage of the insult, sex of offspring, and more. Nutritionally restricted pregnant dams exhibited a reduction in net BW. The net BW of the dam was calculated to show the effect of diet on the dam independently of the growing weight of the fetal/placental unit. Additionally, the dams exhibited a decrease in LRBF and REA beginning at GD 270 suggesting that as the duration of nutritional restriction extends further into the last third of gestation the dam is forced to catabolize adipose and skeletal muscle stores to support the increasing demands of the exponentially growing fetus. Indeed, at this stage of gestation the fetus can grow up to 250 g/d [31].

In the present study, birth weight of calves born to restricted dams was reduced 6.7 kg compared to controls. Similarly, LeMaster et al., [32] observed a 3.8 kg reduction in calf birth weight in response to a similarly modest nutritional restriction over the course of the final 100 days of gestation. After birth, nutrient restricted calves in the present study remained lighter throughout the entirety of the experiment, although a statistical difference was only maintained to PND 105. This may be due to modest levels of compensatory growth as well as an increase in variation due to increasing environmental and genetic influences on postnatal growth. While the ability of calves to exhibit compensatory growth is well established [33], several studies have observed differences in BW of calves born to restricted dams at weaning (~7 months of age) [16, 34] and even at slaughter age [14, 16]. In this study, a single sire was used to minimize genetic variability in the offspring, thus increasing our ability to dissect out potential effects of prenatal nutrition. However, it is important to recognize that the impact of environmental exposures on the offspring phenotype, including birth weight and growth potential, is likely influenced by genetics. Therefore, future studies with larger number of animals will be important to identify the potential interaction between genetics and prenatal nutrition in cattle.

We observed no difference in post-weaning feed intake or gain to feed ratio between groups. However, AUC for glucose following IVGTT on PND 301 and 482 were greater in heifers born from nutrient restricted dams. This is a novel and important finding as, to our knowledge, cattle studies have yet to investigate adult glucose metabolism of progeny born from nutrient restricted dams in late gestation. It is important to note that our findings may reveal a key underlying mechanism that could, in part, explain observations made in previous studies of reduced carcass yield and altered postnatal growth rates following late-gestation maternal nutrient restriction [14, 16, 32, 34]. It remains unclear whether this reduced ability to clear peripheral glucose is the result of reduced insulin production or sensitivity in these heifers. A previous study in our lab, using a similar nutrient restriction model, demonstrated decreased pancreas weights in fetal calves from restricted dams at gestational day 265, along with reduced fetal plasma concentrations of insulin [5]. The pancreases from these fetuses did not show differences in endocrine cell number or location at this time of pregnancy, but the fetal ruminant pancreas has perilobular giant islets, containing mostly beta cells, that atrophy near birth leaving only small islets to persist into adolescent and adult life [5, 35]. While perturbations in the development of the fetal pancreas may be playing a role, there is still a major alteration in energetic efficiency occurring in the adult heifers that warrants further study. Similar results have been reported in retrospective human studies. Women who experienced the Dutch Famine late in pregnancy gave birth to children who exhibited an inability to properly regulate blood glucose as adults [36]. Studies in sheep have also indicated that late-gestation nutrient restriction alters offspring’s postnatal glucose metabolism [29, 37]. Glucose intolerance is one of several risk factors related to the adult onset of type II diabetes. These suboptimal metabolic parameters threaten the postnatal growth and performance of beef progeny. Our findings provide further insight into the underlying metabolic mechanisms controlling economically important production traits.

Visceral fat is a risk factor for reduced insulin signaling in skeletal muscle and adipose tissue [38]. As these are major organs involved in glucose uptake, reduced insulin signaling through its receptor to activate second messengers leads to an impaired ability to clear blood glucose through decreased GLUT4 translocation and utilize glucose within the cell through decreased glucose phosphorylation by hexokinase [39]. High amounts of visceral fat are associated with increased blood concentrations of non-esterified fatty acids (NEFAs), which inhibit activation of insulin receptor substrate-1 (IRS-1), a major regulator of glucose metabolism in the muscle. In the present study, the increased internal (visceral) fat in the heifers born to restricted dams could very well be driving the impaired glucose clearance. Interestingly, subcutaneous fat is also a risk factor for insulin signaling [40], but the thickness of the subcutaneous fat, as measured at the 13^th^ rib, was not different between treatment groups.

Despite not observing differences in whole BW at the time of slaughter, calves born to restricted dams exhibited an increased accumulation of internal fat compared to calves born from well-fed controls. There was no difference in either subcutaneous or intramuscular fat depots between dietary groups. These three adipose depots represent potential for devaluation of the bovine carcass as their quantities are directly utilized to calculate yield and quality grade. From an economic perspective, a 4.2 kg increase in abdominal fat in calves born to restricted dams would be easily offset by the 30% reduction in feed inputs over the last ~125 days of gestation. Nonetheless, these results clearly highlight that energetic efficiency is altered in heifer calves born to modestly nutrient restricted dams. What the implications of an altered energetic efficiency would be had we chosen to retain this heifer in the cow herd remains to be seen. It should also be noted that extrapolation of findings from the present study to male offspring would be inappropriate. A rapidly growing body of literature highlights the relationship between environmental insults during pregnancy, sex of the fetus/offspring, and the observed postnatal phenotype [41]. One relevant example, in sheep, found that a peri-conceptional dietary deficiency in methionine and specific B vitamins resulted in the development of hypertension in male but not female lambs at 9 months of age [42]. A more recent study in pregnant beef cows fed differing levels of protein during the first and second trimesters observed sexual dimorphism in regulation of the thyroid hormone axis associated with differences in milk intake and postnatal growth rates [43].

While muscle tenderness does not have a direct economic impact on the value of the carcass, tenderness is strongly associated with consumer satisfaction and has long-term economic consequences to the beef industry if poor palatability impacts product demand. Underwood et al., [14] detected an increase in muscle fiber diameter and a reduction in meat tenderness in steer calves when cows were nutritionally restricted during mid- to late-gestation. A number of studies have identified a similar increase in muscle fiber diameter when the nutritional restriction is applied from early to mid-gestation [15, 44, 45], likely due to differences in the stage at which the muscle fibers are differentiating during the nutritional insult. In the present study, we found no difference in Warner-Bratzler shear force measures in longissimus dorsi muscles from calves born to nutrient restricted and control fed dams. We also found no difference in marbling score of the longissimus dorsi between groups. These data suggest that a modest late gestation nutritional restriction does not negatively impact carcass quality in heifer calves. It remains to be seen if the differences observed between the present study and the study conducted by Underwood et al., [14] are due to differences in the nutritional paradigm or if this is another sexually dimorphic trait.

A novel and potentially important finding of the present study is that a moderate late gestation nutritional restriction impairs the development of the pituitary gland in the heifer offspring. The pituitary is comprised of the anterior and posterior lobes. Within the anterior lobe are tropic cells that support numerous physiological processes including reproduction, growth, metabolism, lactation, and stress response. Results of the present study indicate that nutrient restriction during late gestation leads to a reduced number of GH-positive cells, somatotrophs, in the pituitaries of heifer offspring. This finding may have major implications in the regulation of the compensatory growth seen in heifers born to nutrient restricted dams as well as the altered glucose regulation and increased adiposity. Previous studies have identified a decreased proportion of somatotrophs in the pituitary of fetal lambs whose mothers were nutrient restricted during mid-gestation [20]. Additionally, growth hormone concentrations are altered in fetal lambs exposed to nutrient restriction as well as children that were intrauterine growth restricted [46, 47]. In sheep, somatotrophs are well differentiated by 115 days of gestation (term: ~147 days) [48]. Metabolic hormones have been shown to regulate apoptosis and cell proliferation of specific cell types in the pituitary [49, 50]. Therefore, it is possible in the present study that changes in nutrient supply during fetal development resulted in metabolic alterations, which, in turn, induced apoptosis of somatotropes and increased proliferation of other cell types in the pituitary. This premise is supported by the observation that the pituitary contains multipotent precursor cells capable of differentiation later in gestation [51]. Alternatively, changes in the number of GH-containing cells in the pituitaries may result from hypothalamic changes in neurons containing GH-releasing hormone (GHRH) and somatostatin, which control GH synthesis/secretion. To our knowledge, this is the first time that an effect of nutrient restriction during gestation on pituitary size and cell proportions in adult offspring has been reported.

GH is involved in stimulating muscle growth, lipolysis, and production of IGF-1 from the liver [52, 53]. These functions have potentially important implications in the production efficiency of cattle that resulted in increased average daily gain and leaner carcasses when GH is exogenously administered [54, 55]. Conversely, GH deficiency results in decreased growth and lean muscle mass as well as alterations in insulin sensitivity and increased visceral fat accumulation in humans and mice [53, 56]. It is possible that the increased visceral fat deposition and altered glucose clearance in heifers from the current study manifest due to a reduction in GH action. That feed to gain ratio was not different in the present study may be due to a lack of statistical power to identify differences in this production parameter or due to compensatory actions of other hormones, such as insulin, to maintain adequate growth.

While this study does not show significant differences in LH-positive gonadotropes, others have shown the pituitary to be a nutritionally sensitive organ with regards to gonadotropin production. Sullivan et al., [57] observed a reduction in circulating FSH concentrations in heifer calves born to cows that had been nutritionally restricted during early to mid-gestation. Sullivan also observed a reduction in the follicular pool of nutritionally restricted heifer calves. Importantly, while there was limited negative impact of our nutritional restriction to PND 485, it remains to be seen how these programmed effects could impact long-term production efficiency if that female was kept in the herd. Martin et al., [19] found that protein supplementation to pregnant cows resulted in heifer progeny that exhibited a 7% higher pregnancy rate in their first breeding compared to heifers born to un-supplemented dams. If these effects are due to functional alterations in the anterior pituitary is currently unknown.

## Conclusions

Results of the present study suggest that a moderate nutrient restriction during the last half of gestation does not significantly impact the production efficiency of heifer offspring to slaughter, and may reduce energy demand and input costs of the cow/calf enterprise. It is important to indicate that results are limited to heifer calves from a single sire and the response of male offspring to an identical nutritional paradigm cannot be inferred from the present study. Results also highlight pituitary gland development and, specifically, somatotrophs, as a possible mechanism for translating gestational nutritional insult into adult alterations in visceral fat deposition and metabolism.

## Supporting information

S1 File Individual values. (XLSX)
